# CRISPRi and beyond: studying essential gene function in the obligate intracellular bacterium *Chlamydia trachomatis*

**DOI:** 10.1128/jb.00059-26

**Published:** 2026-04-22

**Authors:** Adarsh Gopinath, Li Shen, Scot P. Ouellette

**Affiliations:** 1 Department of Pathology, Microbiology, and Immunology, College of Medicine, University of Nebraska Medical Center 12284, Omaha, Nebraska, USA; 2 Department of Microbiology, Immunology, and Parasitology, Louisiana State University Health Sciences Center 12258, New Orleans, Louisiana, USA; National Institutes of Health, Bethesda, Maryland, USA

**Keywords:** *Chlamydia*, CRISPR interference (CRISPRi), gene knockdown, conditional knockout, essential gene, FRAEM, DOPE, small RNA

## Abstract

*Chlamydia trachomatis* is an obligate intracellular bacterium that is the leading cause of bacterial sexually transmitted infections (STIs) and preventable infectious blindness. Its unique biphasic developmental cycle comprises an infectious but non-dividing elementary body and a replicative but non-infectious reticulate body. *C. trachomatis* possesses a reduced genome where more than half of the open reading frames (ORFs) are predicted to code for essential genes, abrogation of which with traditional chromosomal disruption methods is expected to block bacterial growth and developmental cycle progression. However, understanding the function of such genes is critical to expand our knowledge of chlamydial biology and reveal new therapeutic targets. This review aims to compare and contrast four systems developed in the past 5 years for studying essential genes in *Chlamydia*. These include systems to conditionally knock down or knockout a target gene product using CRISPR interference (CRISPRi), inducible small RNAs (sRNA), fluorescence-reported allelic exchange mutagenesis (FRAEM) with inducible complementation of the target gene, and dependence on plasmid expression (DOPE).

## INTRODUCTION

Bacteria belonging to the order Chlamydiales are Gram-negative, obligate intracellular bacteria that can infect a wide variety of hosts, ranging from unicellular amoebae to birds, livestock, and humans. *Chlamydia trachomatis* (Ctr) is a medically significant member of this order that is endemic to humans and can be classified into three major biovars, namely trachoma, genital tract, and lymphogranuloma venereum (LGV). Ctr is subdivided into 19 serotypes based on variations in the major outer membrane protein (MOMP) encoded by *ompA* (1–3). The most widespread diseases caused by Ctr are trachoma and sexually transmitted infections (STIs) (4). Trachoma is the leading cause of preventable infectious blindness worldwide, with up to 190 million people susceptible to this condition (5, 6). Ctr is the leading cause of bacterial STIs in the US, where up to 1.5 million cases are reported every year. However, most infections of the genital tract are asymptomatic, particularly in women, which can lead to chronic sequelae, including pelvic inflammatory disease, infertility, and ectopic pregnancies, when infections are left untreated (7). This suggests that the true burden of chlamydial STIs is underrepresented.

*Chlamydia* possesses a biphasic developmental cycle comprising an infectious but non-replicative elementary body (EB) that attaches to and is endocytosed into a susceptible host cell (8). Inside the host cell, the bacteria reside in a membrane-bound vacuole termed the chlamydial inclusion. Soon after infection, the EB undergoes primary differentiation into the metabolically active, replicative form called the reticulate body (RB) (8). The RB subsequently enters an “exponential” growth phase where they replicate using a unique polarized division mechanism. Recent work indicates that the accumulation of various factors, including oxidizing conditions and c-di-AMP, stochastically and asynchronously triggers secondary differentiation from RB to EB (9, 10). Around 48–72 hpi for Ctr, EBs are released into the extracellular milieu either through extrusion of the inclusion or the lysis of the host cell (11). These infectious progeny initiate a new round of infection.

*Chlamydia* species have undergone genome reduction with smaller genome sizes and gene repertoires than free-living or facultatively intracellular bacteria. The genome of pathogenic chlamydiae contains ~1 million base pairs, with ~900–1,100 open reading frames (ORFs) (12, 13). This is roughly a quarter the size of that of *Escherichia coli*, which has a genome that is 4.6 million base pairs and ~4,400 ORFs (14). This reductive evolution is a result of more than a billion years of host-pathogen interactions that has resulted in a genome that codes for all the major metabolic pathways such as DNA, RNA, protein, carbohydrate, and fatty acid synthesis but generally lacking the genes needed for generating the precursors to these pathways, which are siphoned from the host via transport proteins (13, 15).

Essential genes are defined as those that are absolutely critical for the survival of a cell. In the case of an obligate intracellular pathogen such as *Chlamydia*, this would extend to genes required for invasion and survival within a mammalian host cell, such as the type III secretion system apparatus and the genes involved in the transition of the bacteria between its various developmental stages, such as EB to RB and vice versa. Around 500 essential genes have been predicted to exist for *Chlamydia* (13). This figure (~500) was determined through a pan-genomic analysis of 16 species of the genus *Chlamydia*. A core orthologous group comprising 517 genes was found to be conserved among these strains and linked to critical cellular processes such as translation, ribosome biogenesis, replication, recombination, and DNA repair (13). The ~500 essential ORFs are consistent with the finding that less than 10% of ORFs have been knocked out to date by newly developed genetic techniques and random mutagenesis via transposon or alkylating agents (see below) (16, 17). This number is also consistent with the minimal genome synthetic bacterium, developed by the Ventner Institute, which encoded 473 ORFs; 324 of these were predicted to code for essential biological processes such as cell division, replication, transcription, translation, DNA repair, and membrane biogenesis, while 149 had unknown function (18). Disruption of any essential gene in *Chlamydia* would block developmental cycle progression with severe reductions in infectious progeny production, preventing propagation of a given strain. The absence of axenic media, unlike that developed for the obligate intracellular pathogen *Coxiella burnetii* (19), means that the established chromosomal gene disruption methods commonly used in other systems cannot be used to study these genes without an avenue for the expression of the disrupted gene via a conditional rescue system.

## CHEMICAL AND TRANSPOSON MUTAGENESIS

Until 2011, *Chlamydia* was considered to be genetically intractable relative to model organisms such as *E. coli* or *Bacillus subtilis*. This was attributed to its obligate intracellular dependence and biphasic developmental cycle, making it hard for the delivery and integration of foreign genetic elements. Based on this, different approaches were implemented to create a collection of chemically mutagenized chlamydial strains in an attempt to determine gene essentiality. The first major study employing this approach generated a *C. trachomatis* serovar D (CTD) mutant library using ethyl methane sulfonate (EMS), an alkylating agent that triggers C-G to T-A transition mutations by targeting the nitrogen position at nucleotide bases (20, 21). All bacterial pools containing mutants were plaque-purified and sequence-verified to confirm if the desired mutation was present in the gene of interest, after which the strain was plaque cloned. This approach, otherwise known as targeting-induced local lesions in genomes (TILLING), enabled Kari et al. to isolate a CTD strain containing a null mutation in the β subunit of the tryptophan synthase gene (*trpB*) that was unable to survive IFNγ–induced tryptophan starvation (21, 22).

A 2012 study employed EMS to generate rifampicin-resistant Ctr L2 with multiple mutations per genome and used the resulting mutant library to infect Vero cells for forward genetic screens to identify genes linked to variations in plaque morphology (23). Mutant strains producing several types of granular plaques were picked for whole genome sequencing to establish any common mutations that could potentially have contributed to the aberrant morphology. This was followed by gene linkage studies where rifampicin-resistant granular plaque-producing mutants were co-infected with spectinomycin-resistant WT strains into HeLa cells to encourage the production of double-resistant recombinant strains sporting a smaller subset of the mutations accrued. Recombinant strains exhibiting large or small granular plaque morphology were subjected to whole genome sequencing (WGS) to identify the mutations that strains with similar morphology had in common. Non-synonymous mutations located in *glgB,* a 1,4-α-glucan branching enzyme, and *gspE*, an ATPase required for type II secretion, were associated with the accumulation of insoluble glycogen within the inclusions of Ctr isolated from large and small granular plaques, respectively (24, 25). The *gspE* mutant was later shown to result in a massive decrease in the production of infectious progeny. The most recent major study incorporating a chemical mutagenesis-mediated forward genetic screen involved the generation of 934 Ctr L2 mutant strains expressing the small granular phenotype treated with EMS and a second alkylating agent, N-ethyl-N-nitrosourea (16). The mutations accrued in each strain were characterized via WGS. Kokes et al. determined that 10% of the 894 predicted ORFs in the Ctr L2 genome accumulated null mutants, while up to 62% of ORFs accumulated non-synonymous mutations (16). The bulk of the null mutants were genes associated with varied cellular processes such as metabolism, membrane transport, DNA repair, and transcription, indicating that despite being a part of a reduced genome, some aspects of these processes are either dispensable or have built-in redundancy.

Genetic screens reliant on mutant libraries built via random chemical mutagenesis have played an important role in understanding the molecular biology underpinning chlamydial growth and pathogenesis in a time when targeted manipulation of the chromosome was not possible. However, establishing a link between phenotype and genotype or vice versa is a tedious, labor-intensive process, especially with the lack of selectable markers for the gene of interest. The need for WGS to characterize the often multiple mutations accumulated per genome also makes this process expensive. While there is an outside chance that an essential gene might accumulate favorable hypomorphic mutations that could potentially help the bacteria survive within the host cell in an attenuated manner, the higher propensity for the accumulation of deleterious non-synonymous or null mutations within essential genes will lead to *Chlamydia*‘s being unable to survive and replicate within the host cell. This is bolstered by the fact that most genes discovered through these screens are non-essential for *in vivo* growth and survival.

The first stable transformation of *Chlamydia* has been established based on a CaCl_2_-mediated chemical transformation technique developed by the Clarke lab that relies on a shuttle plasmid (26). The shuttle plasmid is a fusion of the endogenous Ctr plasmid and an exogenous *E. coli* cloning vector. This strategy led to the proliferation of techniques to advance *Chlamydia* reverse and forward genetics (17), including the development of inducible expression systems based on tetracycline-inducible promoters or theophylline-inducible riboswitch-controlled expression (27–29). Recently, transposon mutagenesis has emerged as another tool for *Chlamydia* for the generation of pools of random chromosomal mutants. The goal of labs employing this approach is to achieve saturation mutagenesis (i.e., to generate a mutant pool containing transposon insertion at every possible insertion site present on the chlamydial chromosome). When coupled with a high-throughput screening technique such as transposon-directed insertion site sequencing (TraDIS), it becomes easier to differentiate essential and non-essential genes based on the number of reads accumulated for transposon insertion at a particular site, this number being close to zero for essential genes (30).

The ubiquitous *Himar1* mariner system, comprising a transposon that is cut and paste randomly at single A-T dinucleotide sites independent of any host-specific factors, has been adapted for use in Ctr (31, 32). The first description of a transposon mutant library in *Chlamydia* used a suicide plasmid and included only 105 strains generated across 23 separate experiments, highlighting the low frequency of transformation. Additionally, most mutants did not exhibit a growth defect. Wang et al. adapted this approach for use in *C. muridarum* (33), a mouse strain of *Chlamydia* commonly used for animal studies, and included a GFP reporter to visualize transposon insertions via fluorescence microscopy. Similar to the prior study, the authors ran into a transformation efficiency barrier where only 33 GFP-positive mutants were generated over multiple experiments.

A key issue with both studies, namely low transformation efficiency of the transposon-carrying plasmid, prevented the achievement of saturating mutagenesis. The Clarke group tried to address this by redesigning the plasmid backbone to be self-replicating and regulating the expression of the transposon mutagenesis cassette through the use of an anhydrotetracycline-inducible promoter and riboswitch-controlled expression of the transposon (34, 35). This modified plasmid was used to infect plasmid-free Ctr strains SWFP- and L2(25667R), and the authors isolated 36 and 34 individual transposon insertions, respectively, from each strain. The Clarke lab plasmid was further iterated upon by the Fields lab group to improve its transformation efficiency by reducing its size to better isolate individual strains from complex pools of mutants and wild-type bacteria (36). Using this modified approach, the Fields lab isolated 143 unique strains with insertions distributed across the bacterial genome. Only 15 insertions were found in intergenic regions, with the rest being distributed among operons, CDS, tRNA, rRNA, and pseudogenes. Only 11 genes overlapped with the data published by LaBrie et al., leading to the conclusion that saturation had not been achieved (32). A notable discovery from this study was the isolation of a mutant expressing a truncated inclusion membrane protein *dre1*/*ctl0144,* which resulted in an 8-fold reduction in inclusion-forming unit (IFU) production *in vitro* (36).

Studies to date have made slow but steady progress toward the generation of saturated transposon insertional mutant pools in *Chlamydia* with the express aim of delineating essential genes via complex WGS workflows similar to what has been achieved in genetically tractable and intractable pathogens such as *Salmonella* Typhi and *C. burnetii* (30, 37). However, the obligate intracellular nature of *Chlamydia,* combined with a lack of axenic culture media, means that the chances of isolating a mutant carrying a defect in an essential gene are unlikely. However, data gleaned from such studies can serve as a starting point for labs adopting a targeted genetic approach to study essential genes.

## TOOLS TO STUDY ESSENTIAL GENES IN *CHLAMYDIA*

**Fig 1:**
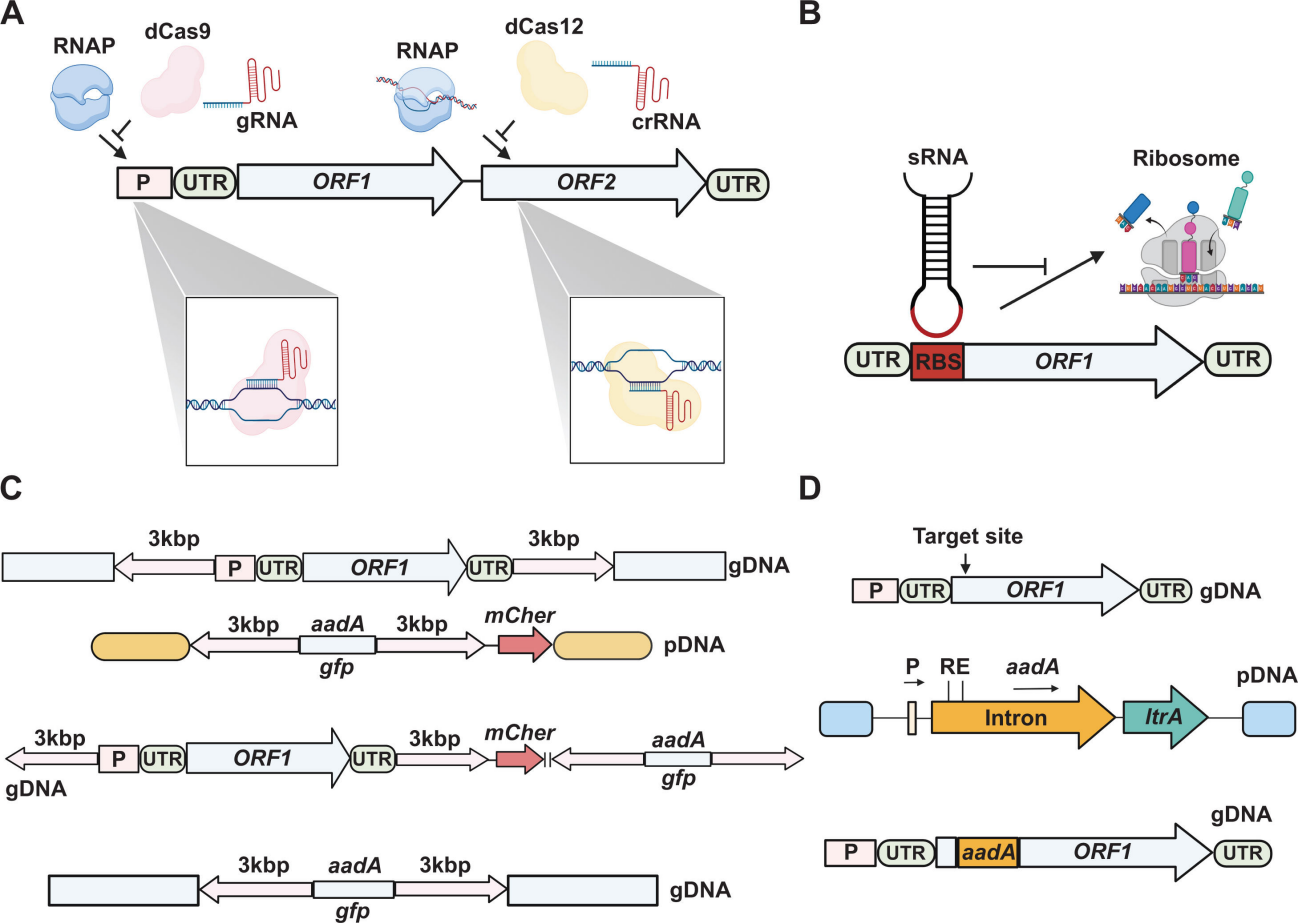
Illustration of methods used to study essential gene function in Ctr. (**A**) CRISPRi-mediated conditional knockdown (KD) strategies target gene expression at the transcriptional level by blocking RNA polymerase (RNAP) binding at a promoter (P) or by blocking RNAP extension within an ORF. A gene-specific gRNA (dCas9) or crRNA (dCas12) is designed to target the ORF and is combined with the inducible expression of the dCas9 or dCas12 protein. (**B**) sRNA-mediated KD strategy targets gene expression at the translational level by binding to the ribosome binding site (RBS) in the 5′ UTR of the target gene’s mRNA. A gene-specific targeting sequence is designed and incorporated into a characterized chlamydial sRNA, the expression of which is inducible. (**C**) FRAEM is used to disrupt the chromosomal allele of a target ORF. A suicide plasmid encoding an *aadA-gfp* selection cassette flanked by 3 kb of genomic DNA flanking both the upstream and downstream segments of the ORF as well as an mCherry cassette elsewhere on the plasmid is transformed into Ctr. After selecting for plasmid integration into the chromosome via a single recombination event to generate a merodiploid, Ctr is transformed with a pSW2 plasmid complementing the ORF under the control of an inducible promoter. After several passages at low MOI while inducing plasmid ORF expression, a second recombination event disrupts the chromosomal ORF with an *aadA-gfp* cassette. A conditional knockout is generated by stopping the inducible expression of the plasmid-based ORF. (**D**) DOPE is conceptually similar to FRAEM but with the steps reversed. First, Ctr is transformed with a plasmid encoding the target ORF under inducible expression. Subsequently, the target ORF is disrupted using group II intron-based (TargeTron) mutagenesis while inducibly expressing the plasmid copy of the ORF. Once the chromosomal allele is knocked out, the function of the target ORF is studied by regulating the expression of its plasmid-based complementing allele. Abbreviations: CRISPR, clustered regularly interspaced short palindromic repeats; CRISPRi, CRISPR interference; crRNA, CRISPR RNA; gRNA, guide RNA; sRNA, small RNA; kbp, kilobase pair; UTR, untranslated region; gDNA, genomic DNA; pDNA, plasmid DNA; *aadA*, encodes spectinomycin resistance; *gfp*, green fluorescent protein; mCher, mCherry fluorescent protein; RE, restriction enzyme cut sites; *ltrA*, reverse transcriptase required for intron insertion. This figure was created in part with Biorender.

This review aims to summarize recent advances in chlamydial molecular genetics systems aimed at studying essential genes through conditional gene knockdown or knockout techniques supplemented with inducible complementation of the target gene. In particular, we will discuss the pros and cons of four major systems: Clustered regularly interspaced palindromic repeat interference (CRISPRi), conditional knockdown of essential genes using small RNA (sRNA), fluorescence-reported allelic exchange mutagenesis (FRAEM) with inducible complementation of target gene, and dependence on plasmid expression (DOPE) (38–41) (Fig. 1; Table 1). A list of all studies using these approaches and the targeted gene is available in Table S1.

**TABLE 1:** List of techniques, including strengths and weaknesses of each approach, used to study essential gene function in *C. trachomatis^a^*

Strategy	Pros	Cons	Studies using this method*
CRISPRi-mediated conditional knockdown (2018/2021)	Simple to execute plasmid-based system, can be multiplexed to simultaneously knockdown multiple genes across the chromosome, little-to-no chance of off-target effects, limited leaky expression (unstable dCas protein, new riboswitch-based systems reliant on induction with two inducers for expression of dCas protein), reversible and tunable, potential for high-throughput, can assess transcripts or protein to validate, "quick"	Polar effects of knockdown in operons when targeting a 5′ gene (can be addressed by complementation with transcriptional fusion of the 3′ gene(s) to dCas gene), need appropriate controls to gauge the effects of overexpression of the dCas protein (easily addressed), potential residual expression of target gene	19*
sRNA-mediated conditional knockdown (2025)	Simple to execute plasmid-based system, possible post-transcriptional knockdown of gene expression, reversible and potentially tunable, "quick"	Transcription of sRNA reliant on leaky aTc-inducible promoter, potential loss of pBOMB plasmid, potential for off-target effects, need appropriate controls to gauge effects of overexpressing a sRNA (i.e., a scrambled sRNA), requires an antibody to validate knockdown, potential for polar effects in operons via Rho-dependent transcriptional termination, potential residual expression of target gene	2*
FRAEM with inducible complementation of target gene (2022)	Conditional knockout of essential genes, uses fluorescence-based readout for recombination that has little-to-no chance for off-target effects, conditional expression of plasmid-based copy, gold-standard approach used in model systems	Time-consuming and low-throughput as gene knockout is reliant on a very low frequency double crossover recombination event, over-expressed proteins from plasmid construct could reduce efficiency of recombination if toxic, expression of target gene could potentially linger for several division cycles after removal of inducer and before effects are measured (can potentially be addressed by making plasmid copy unstable), careful titration of inducer for the complementing allele is needed, potential for mutation of plasmid to allow for constitutive expression of gene of interest, must sequence genome of any strain	1
Dependence on plasmid expression (DOPE) (2024)	Conditional knockout of essential genes, uses group II intron-based system to target chromosomal copy while inducibly expressing a plasmid-based copy	Time-consuming and low-throughput, need for a proprietary algorithm to design targeting sequence, may need to try multiple targeting sequences to knock out a single gene, potential to target plasmid copy instead of chromosomal copy (can modify plasmid sequence to avoid), efficiency of intron integration drops when insert is larger than 2 kb, expression of target gene could potentially linger for several division cycles after removal of inducer and before effects are measured (can potentially be addressed by making plasmid copy unstable), potential for mutation of plasmid to allow for constitutive expression of gene of interest, careful titration of inducer for the complementing allele is needed, must sequence the genome of any strain	1

^*a*^ * includes original description and bioRxiv preprints.

## CRISPRi

CRISPRi was adapted from a naturally occurring prokaryotic adaptive immune system protecting bacteria against viruses and foreign genetic elements (42). While there are many CRISPR systems spread throughout Archaea and Bacteria, the simplest and most well-studied example is that of the Type II CRISPR system encoded by *Streptococcus pyogenes*. It comprises alternating repeating and non-repeating spacer arrays in the bacterial genome that transcribe CRISPR RNA (crRNA), a trans-activating CRISPR RNA (tracrRNA), and a single endonuclease known as CRISPR-associated (Cas) protein 9. The tracrRNA acts as a scaffold that complexes the crRNA with Cas9. The crRNA binds foreign DNA in a site-specific manner, a few base pairs upstream of the protospacer adjacent motif (PAM) specific to the Cas9 protein, which then cuts the invading DNA (43, 44). A synthetic oligonucleotide combining the properties of the tracrRNA and crRNA known as the guide RNA (gRNA) plays an important role in molecular biology-adapted techniques, including CRISPRi.

CRISPRi was the first inducible gene knockdown strategy developed for *Chlamydia* (45). It relies on a catalytically inactive *S. aureus* (Sa) Cas9 lacking its endonuclease activity (known as dead Cas9 or dCas9) in combination with its cognate PAM sequence and a gene-specific gRNA that achieves transcriptional knockdown by sterically inhibiting the binding of RNAP to the promoter region or blocking RNAP elongation on a coding sequence, thus blocking transcription at a specific chromosomal locus. A gRNA is usually designed to target the 5′ intergenic region around the promoter on either the coding or non-coding strand. However, if the target gene is in the middle of an operon, then gRNA sequences should be designed to target the non-template strand of the coding sequence of the gene of interest when using Cas9 (46) (Fig. 1). A proof of concept of a CRISPRi-based conditional knockdown system for *Chlamydia* was published by Ouellette (45) using a single-plasmid anhydrotetracycline (aTc)-inducible dCas9-based system in combination with a constitutively expressed gRNA to knock down the non-essential gene *incA*. IncA facilitates the fusion of individual inclusions within the same cell when infected at high multiplicities of infection (MOI). Loss of function of IncA leads to multiple inclusions within the cell (47). Although this initial system worked to reduce IncA levels (as assessed by immunofluorescence analysis (IFA) of multiple inclusions within cells), there were two principal problems that precluded its use on a population level. The first of these was plasmid instability, which resulted in the presence of mixed bacterial populations with and without resistance to penicillin (expressed from the plasmid). The second issue was leaky expression of dCas9 as a result of the Tet promoter, leading, in some cases, to the inhibition of IncA expression in the absence of aTc inducer.

To address these issues, we made several modifications. First, we switched to a different plasmid backbone. Second, we modified the ribosome binding site of Sa_dCas9 to reduce its translation efficiency. Third, we added a degron to the C-terminus of the Sa_dCas9 protein that would lead to the degradation of any leaky expression of the dCas protein (38, 48). In addition, removal of aTc after inducing dCas expression also leads to reduced dCas protein (and in some instances, depending on the normal developmental expression timing, a recovery in target gene expression). Plasmid stability was verified by tallying the percent of GFP-positive inclusions (GFP is constitutively expressed on the plasmid) relative to the total population (GFP+ and GFP− inclusions), confirming the presence of a relatively pure population of Ctr with limited plasmid loss. Through an IFU assay, an experiment designed to measure the production of infectious EB progeny in Ctr-infected cell cultures, we further demonstrated that the expression of Sa_dCas9 by itself did not have a statistically significant impact on IFU production at any time point or concentration of inducer tested (38). Importantly, the fully reconstituted dCas9-based CRISPRi system targeting *incA* led to no IncA with multiple inclusions per cell, as assessed by IFA as well as reduced *incA* transcripts under inducing conditions only. Therefore, the system could be used for population-level analyses.

The Ctr genome has a GC content of ~42%, and the PAM sequence for Sa_(d)Cas9 is NNGRRT. There are ~32,500 potential PAM sites for Sa_(d)Cas9 in the chromosome of Ctr. To further expand the utility of CRISPRi in *Chlamydia*, we explored the use of a different CRISPRi system based on *Acidaminococcus* dCas12. The cognate PAM sequence of *Acidaminococcus* (d)Cas12, TTTV (where V is any nucleobase but T), is more common (~47,000 sites) compared to the PAM of (d)Cas9 when it comes to the design of gRNAs for the AT-rich genome of Ctr. As with dCas9, a gRNA (called a crRNA for dCas12) is usually designed to target the 5′ intergenic region around the promoter on either the coding or non-coding strand. However, if the target gene is in the middle of an operon, then crRNA sequences should be designed to target the template strand of the coding sequence of the gene of interest when using dCas12. A set of experiments similar to that employed in the assessment of the efficacy of dCas9 was performed for dCas12. Induction of the expression of dCas12 in the presence or absence of a crRNA did not have an impact on the growth and morphology of *Chlamydia* or the inclusion in which it grew. However, at higher aTc concentrations, dCas12 expression alone can reduce IFU production by roughly 2-fold while having limited or no impact on genomic DNA levels or developmentally regulated transcripts, suggesting that it can slightly delay developmental cycle progression (specifically, production of EBs). Knockdown of *incA* transcripts with dCas12 was similarly effective as observed with dCas9, and IFA imaging confirmed that the loss of detectable signal for IncA and the presence of several inclusions per cell were only associated with induction of dCas12 expression in the presence of a gene-specific crRNA. Therefore, both dCas9 and dCas12 systems can be used to inhibit gene expression in *Chlamydia*.

Molecular Koch’s postulates provide a framework for testing the causal role of a gene’s function in a phenotype (49). In many instances, a knockout is generated, and the phenotype is assessed. Subsequently, another copy of the gene of interest (GOI) is introduced to see if a wild-type phenotype is restored by complementation. With the CRISPRi system used in *Chlamydia*, we can fulfill Molecular Koch’s postulates by incorporating a complementing allele into the CRISPRi plasmid backbone. This is achieved by creating a transcriptional fusion of the GOI with dCas9 or dCas12 such that the addition of aTc results in both knockdown of the chromosomal gene product and simultaneous expression of another copy of the GOI. We successfully demonstrated this complementation approach for both CRISPRi systems by transcriptionally fusing a FLAG-tagged *incA* allele to dCas (38). We discuss additional instances below.

The ability to transcriptionally fuse a GOI with dCas also overcomes a major limitation of an approach that relies on blocking RNAP binding or elongation, namely, genes within an operon that are 3′ to the target GOI. Targeting one gene in an operon can result in polar silencing of all genes downstream of the target. To counter this, all genes downstream of the silenced GOI can be transcriptionally fused to the 3′ end of dCas9 or dCas12 to “isolate” the target GOI. Alternatively, each gene in an operon can be targeted to dissect the resulting phenotypes. One example of this strategy was used by Sturd et al. (50) when studying the recruitment and interaction between leucine-rich repeat Flightless-1 interacting protein 1 (LRRF1/LRRFIP1) and its binding partner Flightless 1 (FLI1/FLII) with chlamydial inclusion protein Ct226 and other members of the *ct227* operon (50). The authors employed transcriptional fusions on the 3′ end of dCas12 on the *ct226* KD plasmid with either *ct226*, *ct225*, or *ct224*. Ct226 had previously been shown to interact with LRRF1 (51), and silencing of *ct226* resulted in polar effects that contributed to the simultaneous KD of downstream genes *ct225* and *ct224*. By complementing *ct226*, *ct225*, or *ct224* directly on the *ct226* KD plasmid, Sturd et al. were able to study the individual contribution of each gene toward the recruitment of the host proteins to the inclusion (50).

When targeting an intragenic sequence for knockdown, complementation can still be used to restore expression of the GOI. In one approach, we made synonymous mutations to the gRNA binding and qPCR primer sequences within the *bacA_6xH* gene transcriptionally fused to *Sa_dCas9* (52). This allowed us to measure the knockdown of endogenous *bacA* transcripts while using 6×H labeling by IFA to detect expression of the complementing allele, which restored the wild-type phenotype.

One drawback to this complementation strategy is that transcript levels for the complementing allele under inducing conditions are typically higher than the endogenous levels detected during the developmental cycle (48). If overexpression of the target is detrimental to *Chlamydia*, then this can be problematic, and the effects of overexpression should be compared in parallel to address this (53). We are exploring whether the incorporation of a riboswitch 3′ to the *dCas* gene and 5′ to the complementing allele may allow for reduced and/or tightly controlled expression of the complementing GOI. An alternative would be to incorporate a separate transcriptional unit elsewhere on the plasmid to allow for complementation (see below for sRNA-based approach). However, this would also be complicated either by gene dosage effects, if using the endogenous promoter sequence for the target gene, or potential recombination within the plasmid, if using the Tet promoter.

The impetus for developing the CRISPRi system was to have a means to study essential gene function in *Chlamydia*. To that end, it has been used by us and others in a variety of contexts to study critical functions of the bacterium (Table S1). We note that in our experimental approaches, we induce knockdown prior to the peak transcription of the target gene to limit the possibility that the stability of the target protein masks the effects of knockdown. Wood et al. (54) used CRISPRi to demonstrate the essentiality of the ClpXP protease in *Chlamydia*. Reduced *clpP2* and *clpX* transcripts were associated with significantly reduced IFU output that was partially restored by *clpP2* complementation. In similar studies, the essentiality of the ClpCP protease was also demonstrated (55). Both the ClpXP and ClpCP proteases are typically dispensable in many free-living bacteria, thus highlighting how genes that are non-essential in most organisms can be essential in *Chlamydia*. Other CRISPRi studies have demonstrated the essentiality of key genes involved in replication/transcription (e.g., *topA* [53]), translation (e.g., *obgE* [56]), cell division (e.g., *ftsK* and *pbp2* [57]), metabolism (e.g., *ahpC* [10]), and signaling (e.g., *dacA* [9]).

In another demonstration of the efficacy of CRISPRi, Wood et al. (48) employed CRISPRi to knock down *tmRNA*, a tRNA/mRNA hybrid RNA species that forms a part of the *trans*-translation protein degradation system. *tmRNA* works by displacing an mRNA template from a ribosome stalled on a non-stop mRNA. The ribosome is able to translate the tmRNA sequence, which adds an SsrA degradation tag to the stalled translation product that leads to degradation of the incomplete protein (58, 59). Greater than 90% knockdown of *tmRNA* transcripts was achieved through a gRNA targeting its promoter region. Knockdown of *tmRNA* transcripts resulted in disruption of the developmental cycle with smaller inclusions, reduced replication, and decreased IFU output. Knockdown of *tmRNA* was complemented by expression of a wild-type *tmRNA* driven by the *incD* promoter expressed elsewhere on the KD plasmid to restore *tmRNA* expression during induction of dCas9. The complementation approach was necessarily different in this context to ensure that the complementing RNA was expressed in its native form and not as a transcriptional fusion with *dCas9*. This study highlights the utility of CRISPRi in blocking the expression of RNA-encoding genes in addition to protein-encoding genes.

Recently, we demonstrated an additional capability of the dCas12-based knockdown system, specifically its capacity for multiplexed knockdown. The dCas12 CRISPRi system in its current state can be modified to express multiple crRNAs targeting different genes simultaneously. This act of multiplexing crRNA was first reported by Hatch & Ouellette (60) in their study to identify alternative sigma factor regulons. crRNAs targeting the 5’ promoter region of σ^28^, σ^54^, and *incA* were inserted into the dCas12 encoding plasmid pBOMBL12CRia either separately or in combinations of two or three crRNAs per plasmid. Knockdown efficiency of the target gene products in the double and triple KD strain was similar to when a plasmid expressing a single crRNA was used. The major drawback of this approach is the cloning of the crRNAs into the plasmid, as gene synthesis does not currently accommodate multiple tandem repeat regions (here, the crRNA scaffold) within a short sequence length. Nevertheless, multiplexed knockdown should further enhance our ability to study gene networks in *Chlamydia*.

There are some potential concerns in using CRISPRi in *Chlamydia*. Although we have designed the CRISPRi plasmids to limit leaky expression of the Tet promoter-controlled *dCas* gene under non-inducing conditions, it is likely that some dCas protein may be stochastically expressed (even if ultimately degraded through its degron). For a gene target that is essential and expressed at a low level, this could be problematic as, in theory, only one dCas protein in combination with its gRNA is needed to block RNAP function. We have encountered a few instances where we were unable to isolate a chlamydial transformant consistent with this (unpublished observation). To address this, we have introduced a synthetic E riboswitch downstream of the aTc-inducible promoter as described and implemented by the Grieshaber lab (29). The E riboswitch has an aptamer that binds theophylline and undergoes a conformational change that allows ribosome access to the otherwise occluded ribosome binding site. Hence, for the dCas protein to be expressed, two inducers at suitable concentrations are now needed, namely aTc and theophylline.

Although we have not encountered issues of plasmid stability under non-inducing conditions, we have observed plasmid loss after inducing knockdown from our original CRISPRi vectors. This is not necessarily a problem, as it further highlights the essentiality of the target and can be quantified (54). However, because penicillin (the selection agent in our original vectors) is not truly bactericidal to *Chlamydia* in the way it is for other bacteria, organisms can lose the plasmid, become susceptible to penicillin, and produce aberrant inclusions, which are detectable and quantifiable. This can potentially complicate population-level analyses. Part of the reason for plasmid loss in the pBOMB-derived vectors is the presence of a repeated sequence element at the junctions of the shuttle plasmid (i.e., between the Ctr and *E. coli* plasmids). To address this, we have created second-generation CRISPRi vectors that lack one of these repeated elements and also encode spectinomycin resistance (*aadA*). Spectinomycin at higher concentrations is bactericidal to *Chlamydia*. This combination has led to no detectable plasmid loss under the conditions we have tested (unpublished observation).

Although off-target effects can be a concern in eukaryotic systems when using CRISPR strategies, the small genome of Ctr limits this. Our typical gRNA or crRNA design shows no more than 14 bp homology in a 21 bp targeting sequence, meaning that there exist seven or more mismatches. This is close to random, and we have not observed instances of off-target effects in Ctr. We make one final note about the level of knockdown typically observed. In all of the CRISPRi studies published to date in the field of chlamydial genetics, the highest rate of KD of a target gene transcript was ~90% (10), with typical knockdown levels between 75% and 90%. For highly transcribed genes, the residual amount of transcripts not targeted might still be enough to mask the effects of targeted gene knockdown and not reveal a phenotype. However, we suggest that some of this residual transcript may arise from inert particles present in a chlamydial stock that remain bound to cells or reflect non-productive infections. Whenever purifying and isolating chlamydiae, a significant portion of isolated EBs can be non-infectious or otherwise defective (61). This may lead to an underestimation of knockdown efficacy. Regardless of whether knockdown is “complete,” if phenotypes are measurable and reproducible and can be complemented, then the efficiency of knockdown is irrelevant.

Overall, CRISPRi has allowed rapid advances in our understanding of fundamental chlamydial biology. Looking forward, we are excited by the possibility of combining CRISPRi strategies with gene knockout strategies to reveal conditional lethality and other interacting gene networks.

## CONDITIONAL KNOCKDOWN USING SMALL RNA (SRNA)

The Sütterlin group in 2025 described a method to inducibly knock down target gene products through the use of an sRNA that targets the ribosome binding site (RBS) in the 5′ untranslated region (UTR) of a GOI (41). Binding of sRNA to the mRNA at this particular site is expected to prevent the recruitment of ribosomes and in turn reduce translation of the target message and levels of its encoded protein. In theory, this system constitutes a post-transcriptional mechanism of knockdown, but we comment further on this below. The sRNA used in the study was based on CtrR3, an sRNA that is constitutively expressed during the Ctr developmental cycle (62). The targeting sRNA is designed based on rules established for the design of *E. coli* antisense RNA (63). In brief, the putative RBS for the gene of interest was identified by visually scanning for GC-rich sequences exhibiting similarity to the Shine-Dalgarno sequence in the 5' vicinity of the start codon. Two or more 30-nucleotide sequences were then analyzed for target specificity via TARGETRNA3 (64). The favored sRNA sequence was then inserted into the single loop of CtrR3, and the conformation of the resulting construct was verified through RNAFold (65). The modified CtrR3 sequence is then cloned into pBOMB5-tet-CtrR3 plasmid, under control of an aTc-inducible promoter, and transformed into Ctr via the CaCl_2_-mediated chemical transformation protocol (26). While, in principle, the 30 nucleotide sequence should limit any off-target effects, it is not clear if all 30 nucleotides bind the target sequence or if only a subset does. If the latter, then the potential similarity of ribosome binding sites in the genome suggests that off-target effects could occur, and future work should more carefully assess this.

Using the above technique, the authors inducibly knocked down the translation of inclusion membrane protein IncA and, for the first time, the Major Outer Membrane Protein (MOMP), the latter of which resulted in inclusions containing a handful of enlarged RBs and a 16-fold reduction in the production of infectious progeny relative to uninduced samples. Knockdown of IncA resulted in the well-established multi-inclusion phenotype observed in Ctr strains lacking *incA* (47). The wild-type phenotype for IncA knockdown was subsequently rescued through aTc washout or complementation of *incA* on the same plasmid under the control of a different Tet promoter. Note that, in this system, similar to complementing *tmRNA* knockdown as described above (48), complementation requires a distinct copy of the allele encoded elsewhere in the plasmid to allow the sRNA to be expressed intact. The drawback in using another Tet promoter is the possibility of recombination within the plasmid or increased leakiness from both promoters by titrating TetR levels. We also note that the pBOMB5 vector backbone likely contains the same repeated regions at the junctions of the shuttle plasmid as described above in our original CRISPRi vectors that can lead to plasmid loss. To our knowledge, the authors did not assess plasmid loss in their study.

The authors next chose to knock down the products of *incE* and *incG* of the *incDEFG* operon to demonstrate the technique’s ability to target individual genes of an operon. While western blot analysis and IFA were used to quantify depletion of the target protein, RT-qPCR analysis revealed transcriptional effects associated with sRNA-mRNA interactions, namely the reduction of *incE* and *incG* transcripts by ~50% relative to the uninduced sample in the IncG knockdown strain. No data were presented to assess transcript levels in the IncE knockdown strain, but the authors did note similar reductions in *incA* transcripts in the IncA knockdown strain. Blocking ribosome initiation or translation (i.e., stalling) can lead to Rho-dependent polarity and premature transcription termination (66), and we have previously demonstrated these effects in *Chlamydia* (67). The authors were perhaps fortunate in assessing an operon with small genes, as it is likely that in larger genes, polar effects would be more pronounced. We would recommend that others using this strategy in such contexts carefully assess transcript levels of the target gene and any genes 3′ to the target gene in an operon.

Besides likely impacts on transcript levels, the major drawback of this strategy is that it relies on antibodies, or potentially mass spectrometry analysis, to measure knockdown of the target gene product. Antibodies for most genes of interest in Ctr are not commercially available, and this was demonstrated in the sRNA study by the lack of antibodies to determine the effects of IncE or IncG knockdown on IncD and IncF levels. It is well established that Tet-inducible promoters are leaky by nature, and hence, there will likely be basal expression of the sRNA and the complemented rescue protein, and this could lead to the potential knockdown of the protein of interest even in the absence of the inducer. Unlike with dCas9 or dCas12, expression of the sRNA cannot be regulated by the addition of a theophylline-inducible riboswitch. Finally, we would recommend including a non-specific scrambled RNA negative control of similar genetic composition as the sRNA being tested to study the non-specific effects of overexpressing sRNA off a plasmid and also to offer a baseline to compare with the gene-specific sRNA. Overall, this approach may complement other strategies to aid in the study of essential gene function in *Chlamydia*.

## FLUORESCENCE-REPORTED ALLELIC EXCHANGE MUTAGENESIS WITH INDUCIBLE COMPLEMENTATION OF TARGET GENE

FRAEM was the first technique developed for *Chlamydia* that could modify large segments of DNA via homologous recombination allelic exchange without the size constraints associated with group II intron insertions (68). A major innovation in this study was the development of a Ctr pL2-based suicide vector expressing mCherry that relied on the alteration of plasmid stability through the Tet-inducible expression of *pgp6*, encoding a plasmid maintenance protein, where removal of the inducer resulted in the loss of plasmid. Through the use of this plasmid, Mueller et al. (68) were able to delete *trpA*, encoding the α subunit of tryptophan synthase, through allelic exchange with a construct comprising green fluorescent protein (GFP) and β-lactamase sharing 3 kb flanking sequences with *trpA*. Allelic exchange and loss of plasmid were tracked through fluorescence microscopy. After successful gene deletion and loss of suicide vector as indicated by GFP+ only bacteria, a second plasmid complementing *trpA* and expressing *aadA* (Spectinomycin resistance) was transformed to successfully rescue the lost gene function. In the same study, the authors deleted the type III secretion system effectors CTL0063, CTL0064, and CTL0065.

Cortina et al. (39) used FRAEM to facilitate the deletion of the presumably essential gene CTL0402/*incS*. They achieved this through a four-step process. The first step was the design of the pSU-Δ*incS* plasmid containing an *aadA-gfp* selection cassette flanked by 3 kb of genomic DNA flanking both the upstream and downstream segments of *incS*. This plasmid also constitutively expresses mCherry and encodes *pgp6* under the control of the Tet promoter on the backbone. This plasmid was transformed into WT Ctr L2 in the presence of spectinomycin and 50 ng/mL aTc (to maintain plasmid) to obtain a population of GFP- and mCherry-positive bacteria. Subsequently, the transformed bacteria were passaged multiple times at low MOI (0.2) to select for mutants expressing faint GFP and mCherry, indicating that a single recombination had occurred with the plasmid being inserted within the flanking 3 kb up or downstream of the WT gene. The third step was to transform the complementation plasmid pSW2-TetIncS_Ct_ encoding a 3×FLAG-tagged *incS* under the control of the aTc-inducible promoter. Ctr was passaged in media containing low levels of aTc (0.5 ng/mL, empirically determined) to induce low-level expression of *incS_3xflag*. The final step was to subject the transformed Ctr strains to multiple passages at low MOI (0.2) in the presence of aTc, spectinomycin, and penicillin (*bla* encoded on the complementation plasmid). This eventually resulted in a second crossover event in some bacteria, leading to the allelic replacement of the WT *incS* ORF with an *aadA-gfp* cassette and the loss of mCherry expression. Once half the bacterial population was GFP-positive and mCherry-negative, *Chlamydia* was plaque-purified, and the resulting mutant was named Δ*incS* Ct. In the absence of an aTc inducer to drive expression of the plasmid-derived *incS_3xFLAG*, Δ*incS* Ct displayed a 1.5 log reduction in EBs relative to WT and the complemented strain. Further experiments indicated that IncS plays an important role in the transition from EB to RB and is essential for the development of Ctr in the vaginal vault of mice.

The major advantage of this type of strategy is that a true conditional knockout can be generated by removing aTc from the culture. However, the leaky nature of Tet promoters suggests that, even under such conditions, small amounts of the protein may be expressed. Again, this is irrelevant if reproducible phenotypes are measured in the absence of detectable protein or significantly reduced transcripts. The major drawback is that the entire process is extremely time-consuming and relies on two low-frequency recombination events. After the final step, the entire genome of the transformed chlamydial strain should be validated via whole genome sequencing to verify if allelic exchange was restricted to the target gene. On top of being a time-consuming process, there are several other issues that could potentially limit the effectiveness of this method. Given that the initial transformation uses a plasmid with 3 kb DNA flanking regions, there will be an increase in gene dosage for any encoded proteins within the flanking regions. This could be problematic in some contexts, and the efficiency of recombination or the initial transformation could be reduced if the “over-expressed” protein from the plasmid is detrimental to chlamydial growth. The complemented target gene could potentially linger for several division cycles after removal of the inducer and before effects are measured, but this could be addressed by making the plasmid copy of the gene unstable by adding an SsrA degradation tag to the complemented protein (48). Careful titration of the inducer for the complementing allele is needed as too high a concentration of the inducer could result in the overexpression of the complemented protein. Given the number of passages, there is always the potential for mutation of the plasmid to allow for constitutive expression of the gene of interest, preventing the modulation of gene expression through the removal or addition of an inducer. Overall, this strategy is closest to techniques used in more traditional model systems and represents an ideal means to create a conditional knockout.

## DEPENDENCE ON PLASMID EXPRESSION

DOPE-based conditional knockout of essential genes relies on a group II intron-based system known as TargeTron (Sigma Aldrich, St. Louis, MO) to disrupt a gene of interest with an antibiotic resistance cassette while using a shuttle vector to conditionally express the target gene under the control of a Tet promoter (40). This approach is conceptually similar to the FRAEM approach developed by the Derré lab (39). Withdrawal of the aTc should deplete the complemented gene product, thus allowing for a conditional knockout. TargeTron-based gene knockout relies on proprietary algorithms to generate gene-specific group II introns. Group II introns are self-splicing ribozymes that are capable of high-frequency movement between genes through a process known as retro homing. It relies on its intron-encoded protein for its reverse transcription, mRNA splicing, and conformational change to move from one location on the genome to another. Johnson and Fisher (69) were the first group to adapt this system in *Chlamydia* by disrupting the *incA* gene with a β-lactamase cassette. The Fan lab built upon this work to develop DOPE to target the essential chlamydial transcriptional regulatory protein GrgA (40).

Lu et al. (40) designed a group II intron containing *aadA* to insert between nucleotides 67 and 68 of *grgA*. To facilitate this, they built their gene-specific group II intron plasmid around a previously characterized TargeTron suicide vector developed by the Fisher lab, pDFTT3(aadA), and named it pDFTT3(aadA)*grgA*-67 (70). However, the first step required transforming Ctr L2 with a shuttle vector expressing a Tet-inducible *grgA* allele with a 6×His tag and four synonymous point mutations, making it resistant to the *grgA* group II intron, and a weakened ribosome binding site assembled on a pTLR2 shuttle vector (pGrgA-DOPE). The resulting strain was called L2/cg-peig. After ensuring the induction of the modified *grgA* did not have an impact on bacterial growth or on the production of infectious progeny, pDFTT3(aadA)grgA-67 was then transformed into L2/cg-peig and incubated with spectinomycin to select for Ctr mutants carrying a disrupted *grgA* chromosomal allele, now named L2/cgad-peig. In subsequent experiments, GrgA-deficient Ctr exhibited reduced RB growth and next to no EBs when sampled 24 h post-removal of aTc and fewer than 300 EBs after 36 h. Depletion of GrgA also resulted in the downregulation of late genes, the target genes of σ^28^, σ^54^, and Pgp4, and the disruption of the midcycle transcriptome. Of note, a portion of the progeny EBs accumulated mutations in *tetR* and started expressing *grgA* via DOPE even in the absence of aTc, suggesting that population-wide studies could be compromised. In addition, the authors did not send their L2/cgad-peig strain for whole genome sequencing to verify if the TargeTron had any offsite insertions, instead relying on Sanger sequencing around the target site. While DOPE is relatively faster than FRAEM when it comes to conditional gene knockout, it still suffers from a few cons associated with the use of the TargeTron platform and complementation of the target gene.

TargeTron is a proprietary system where one buys access to the design algorithm. Group II intron insertion efficiency is variable and requires the design of introns targeting multiple insertion sites (71). The efficiency of intron integration drops even further if the insert size exceeds 2 kb in size (72). As with other approaches, disruption of a gene in an operon can lead to polar effects. One of the downsides of complemented gene expression is the chance that the protein product might linger for several cell division cycles even after the removal of the inducer. This could potentially be alleviated with a degradation tag. The authors did not use this strategy for *grgA* but noted a rapid decline in GrgA levels after removing aTc, perhaps suggesting inherent instability of the protein. A second downside of complemented gene expression is an issue the authors themselves acknowledged, namely the accumulation of mutations in *tetR* or tetO (TetR operator), leading to constitutive expression of the target gene in some progeny EB. This could potentially affect population-wide studies such as the analysis of qPCR or transcriptome data. The authors may be able to address this, as well as leaky expression from the Tet promoter, by including a riboswitch downstream of the promoter to require two levels of induction to induce gene expression. A third downside is the requirement for careful titration of the inducer for the complementing allele. With the need to modify the complemented allele from being susceptible to the TargeTron in use and the need for whole genome sequencing to confirm lack of off-site insertion, DOPE is a time-consuming process relative to the CRISPRi approach but relatively faster than the FRAEM strategy described above.

## CONCLUSION

The development of a means to stably transform Ctr with a plasmid has led to rapid progress in our ability to study this obligate intracellular bacterium (26). The nature of the biphasic chlamydial developmental cycle, coupled with a reduced genome likely rendering most genes essential, makes the determination of the molecular mechanisms underpinning cell division, differentiation, and pathogenesis extremely difficult. Recent advances in developing methods, described here, to conditionally knock down or knockout gene expression will aid in the study of essential genes. Related to this, the small size of the chlamydial genome represents both a challenge and an opportunity. Developing libraries to target every gene for knockdown or knockout is feasible with community support and will significantly aid the field in deciphering the unique biology of *Chlamydia*.

## SUPPLEMENTAL MATERIAL

Table 1 jb.00059-26-s0001.xlsx Works utilizing CRISPRi.

ASM does not own the copyrights to Supplemental Material that may be linked to, or accessed through, an article. The authors have granted ASM a non-exclusive, world-wide license to publish the Supplemental Material files. Please contact the corresponding author directly for reuse.
